# Enriched environments enhance cognition, exploratory behaviour and brain physiological functions of *Sparus aurata*

**DOI:** 10.1038/s41598-020-68306-6

**Published:** 2020-07-09

**Authors:** P. Arechavala-Lopez, J. C. Caballero-Froilán, M. Jiménez-García, X. Capó, S. Tejada, J. L. Saraiva, A. Sureda, D. Moranta

**Affiliations:** 1 Fish Ethology and Welfare Group, CCMAR, Faro, Portugal; 2 Fish Ecology Group, IMEDEA (CSIC/UIB), Esporles, Spain; 3 Laboratory of Neurophisiology, Universitat de les Illes Balears (UIB), Palma de Mallorca, Spain; 4 Research Group in Community Nutrition and Oxidative Stress, University of Balearic Islands and Health Research Institute of the Balearic Islands (IdISBa), Palma de Mallorca, Spain; 5 CIBEROBN (Physiopathology of Obesity and Nutrition), Palma de Mallorca, Spain

**Keywords:** Biochemistry, Physiology, Zoology

## Abstract

Environmental enrichment is considered as a recommended tool to guarantee or improve the welfare of captive fish. This study demonstrates for the first time that structural environmental enrichment enhances cognition, exploratory behaviour and brain physiological functions of gilthead seabream (*Sparus aurata*). Seabream was reared in groups (n = 15) during 60 days under two different treatments: enriched tanks with plant-fibre ropes (EE) or bare/non-enriched tanks (NE). Fish were then exposed to a purpose-built maze for 1 h every second day in four trials. Analysis of video recordings showed that seabream under EE conditions presented higher overall exploratory behaviour, spatial orientation and learning capability compared to seabream from NE conditions. Results from brain monoamines analyses may suggest increased recent dopaminergic activity in telencephalon, known to be involved in learning processes; and increased serotonergic activity in cerebellum, involved in the coordination of balance, movements and orientation. In addition, EE-reared fish showed increased antioxidant activity in whole brain, with no apparent oxidative damage. Structural EE seemed to induce an hormetic response on juvenile seabream, improving their welfare status during captivity. Application of this kind of physical structure might be feasible at fish farms as a passive and non-invasive tool to improve welfare of intensively cultured seabream.

## Introduction

In natural environments, stress responses have evolved to help the animal survive to challenges of various types. However, natural stressors tend to be brief and/or avoidable, while stressors of anthropogenic origin in captivity may be unavoidable and prolonged or repetitive. Under such circumstances, chronic or repeated activation of behavioural and physiological stress responses is not adaptive and can be detrimental to the animal. The interest in studying stress and welfare of captive fish has recently increased to address the negative impacts associated with rearing fish in captivity worldwide^1,2^, and especially in intensive aquaculture^3^. However, this interest has expanded into recognising the importance of positive welfare (i.e., mental and physical states that exceed what is strictly necessary for short-term survival; see^4^ for a review). Environmental enrichment (EE) is considered as a recommended tool to guarantee or improve the welfare of captive fish^5^. The deliberate addition of physical complexity to captive conditions allows the animals to have a greater control over their environment, and provides the opportunity to experience new situations while performing behaviours typical of their species in the wild^5^. Well-designed structural EE may provide sensorial and motor stimulation that meet the animals’ behavioural and psychological needs, while increasing the behavioural options and putatively reducing the stressors. Captive environments generally lack structures, mainly due to practical reasons and biosecurity, and there are several procedures that can be very stressful for fish during a cycle of aquaculture production, such as handling, crowding, vaccination and transportation among others^6^. Environmental complexity, made up of physical structures (stones, roots, logs, plants, algae, sand, sessile animals, ice, artificial objects, etc.) is an important environmental factor in the natural environment of many species, and can thus mitigate environmental stressors in captivity, such as human activity or intraspecific aggression, improving the welfare status of captive fish (for a review see^7^).

Previous studies showed that complex enriched environments improve behavioural flexibility and cognitive ability of fish, enhance the adaptability to novel situations and learning, and stimulate cell proliferation in diverse brain regions known to be involved in these processes (e.g.^8–12^.). Some of the main modulators of such behavioural and brain functions, including learning, memory, cognition, motor processes, stress responses and emotional states, are monoaminergic neurotransmitters^13–17^. Therefore, brain monoaminergic activity, measured by levels of monoamines and their precursors, may be a reliable indicator of fish welfare. Another relevant aspect of introducing physical structures in captive environments is the modification of fish activity. Adding physical complexity potentially increases sheltering, alters aggressive actions, reduces stressful situations and promotes exploratory behaviour^7^. Changes in swimming behaviour could reflect how a fish is sensing and responding to its environment, which might be reflected in its ventilatory rate and oxygen consumption^18^. Higher swimming activity leads to a higher ventilatory activity, ensuring the supply of oxygen at the exact rate required by cellular oxygen metabolism, which is regulated primarily to avoid oxidative stress at the cellular level. In order to maintain the redox cell state in equilibrium, fish may reduce the reactive oxygen species (ROS) through the action of specific antioxidant enzymes, such as catalase (CAT), superoxide dismutase (SOD), glutathione peroxidase (GPX) and glutathione reductase (GRd). Levels of malondialdehyde (MDA) are used as markers of oxidation of membrane phospholipids through lipid peroxidation. As for antioxidant enzyme activities, oxidative damages have been used in aquatic organisms as biomarkers of oxidative stress^19^, and therefore, might be used to better-understand the swimming activity and related welfare status of captive fish.

The aim of this study was, therefore, to assess the effects of structural EE on welfare of juvenile sea bream (*Sparus aurata*) under experimental conditions, namely in spatial cognition (exploratory behaviour and spatial learning abilities) and neurophysiological indicators, such as monoaminergic activity in different brain regions and brain oxidative stress levels. The addition of structural elements into rearing environments might evoke generally positive brain and behavioural responses, and therefore, an overall improvement in health and well-being of captive fish.

## Material and methods

### Fish and experimental design

Gilthead sea bream juveniles (n = 90; mean standard length: S.L. ± SE = 9.3 ± 0.1 cm; mean body weight: TW ± SE = 21.9 ± 0.8 g) were obtained from a commercial hatchery (Aqüicultura Balear S.A./Culmarex Group, Mallorca, Spain) and acclimated to the laboratory conditions in quarantine tanks for 30 days, before being randomly distributed to six 150 L rearing tanks (initial densities 5 kg m^−3^; 15 fish tank^−1^). Three tanks were enriched with five plant-fibre ropes hanging from the top, equally-distant to each other. Both structural enriched and non-enriched tanks (hereafter EE and NE tanks respectively) had a semi-open flow seawater system, provided with mechanical filters, UV sterilisation and compressed air supply^20^. The photoperiod was operated on a 12:12 h light/dark cycle and water quality was checked daily: temperature was 20 ± 1 °C degrees throughout all the experiment, salinity was 38 ppm and dissolved oxygen was kept close to saturation by aeration through diffusion stones^20^. The tank was thoroughly cleaned daily by siphoning waste settlement (faeces and uneaten pellets). Fish were maintained under these experimental conditions for 60 days (30/05/2018–31/07/2018). They were daily fed by hand at 9:00 a.m. a commercial pelleted diet (sinking pellets; 2% of their body mass) specific for gilthead seabream (Skretting, Nutreco N.V., The Netherland). At the beginning (t_0_) and at the end (t_60_) of the experimental period, all fish were length measured (SL) and weighed (TW). Fulton´s condition factor (K = 100*TW*SL^−3^) and the increment of biometric measures were estimated. All the procedures with fish were approved by the Ethical Committee of Animal Experimentation (CEEA Ref. 85/02/18) and carried out strictly by trained and competent personal, in accordance with the European Directive (2010/63/UE) and Spanish Royal Decree (RD53/2013) to ensure good practices for animal care, health, and welfare.

### Behavioural experiment

**Figure 1:**
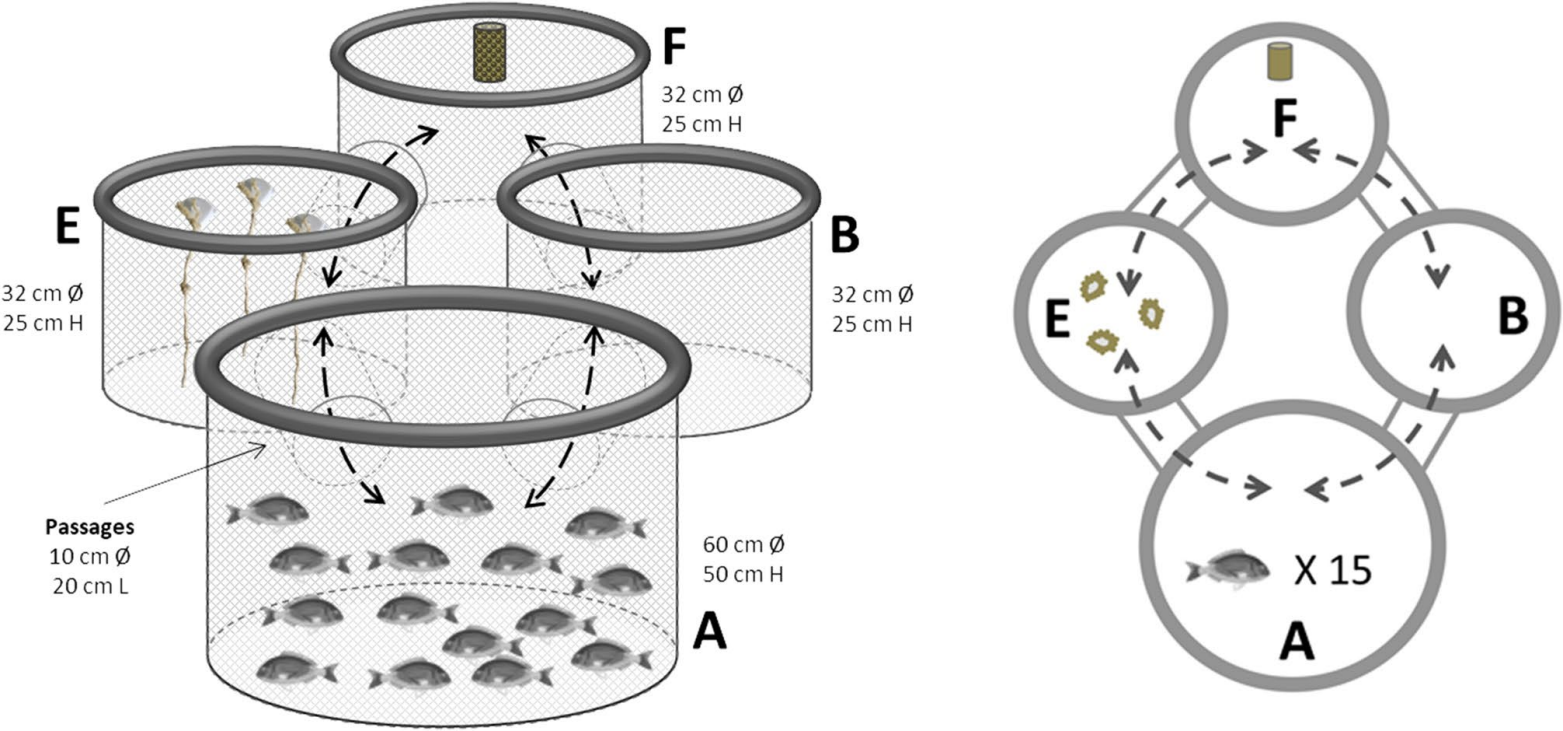
Schemes of the new-designed fish maze: four floating circular cages made by plastic net with foam rings, connected among them through tubular passages (10 cm diameter × 20 cm length). Cage A: 60 cm diameter × 50 cm depth; cages B (bared), E (enriched) and F (feed): 32 cm diameter × 25 cm depth. Fish group (n = 15) started in cage A and behavioural parameters were recorded for 1 h. This test was repeated during four consecutive days and for all fish groups. Dashed arrows show connectivity between cages. Note that cage A and F are not directly connected, only through E or B.

In order to assess potential effects of EE on fish behaviour, a maze experiment was carried out on every juvenile seabream group (15 fish from each rearing tank and three tanks from each experimental condition) during four trials at days 51 (t_51_), 54(t_54_), 56 (t_56_)and 58 (t_58_), counting from the beginning of the experiment(enrichment/non-enrichment exposure). Every fish group was moved into a new-designed experimental maze and video-recorded from above for 1 h. The maze consisted of four floating cylindrical cages, made of plastic net and foam rings, connected among them (Fig. 1). The maze was located inside a larger tank of 10,000 L with same water system and similar environmental conditions as previously. Fish were placed into the initial cage (cage A: 60 cm Ø, 50 cm depth), which is connected by tubular passages (10 cm Ø; 20 cm length) to two smaller cages (32 cm Ø, 25 cm depth): one with enriched structures (cage E) and another one completely bared (cage B). The enrichment in cage E consists of three plant-fibre ropes attached equally distant to the bottom of each cage with two nots alongside and a little buoy on top to keep them in vertical position^20^. Likewise, both cages (E and B) were respectively connected by tubular passages (10 cm Ø; 20 cm length) to cage F (32 cm Ø, 25 cm depth), where a pierced bottle full of food pellets was hanging at the most distant point (Fig. 1). The concept of this purpose-built maze was based on commonly used T-maze and two-chambers apparatus to assess fish learning, but also on group-based risk-taking tests previously developed on seabream^21–23^. The maze allows the assessment of spatial cognition through examining the exploratory behaviour of fish in a novel area and, as well as the spatial learning process of fish throughout the four-day trials of the experiment. Fish were recorded for 1 h and afterwards all fish were placed back into each experimental rearing tank. All recorded videos were visually analysed and the following behavioural parameters registered to compare between treatments and among tanks: i) latency of first individual to leave area A and appear in area E (T_A→E_) or in area B (T_A→B_); ii) latency of first individual to appear in area F coming either from area E (T_E→F_) or from B (T_B→F_); and iii) latency of first individual to bite the food in area F (T_Feed_). Maximum latency considered was 60 min (end of observation). Additionally, the frequency of fish movements between two areas were also recorded (N_A↔E_; N_A↔B_; N_E↔F_; N_B↔F_) as a proxy of fish activity and spatial orientation. All behavioural parameters were also analysed over time (4 days) to assess the learning capability and behavioural plasticity of seabream.

### Neurophysiological analysis

Once the experiment ended (day 60), all fish were euthanized by overdoses of tricaine methanesulfonate (MS-222; 0.1 g/l; 15 min). Then, brain samples from 10 fish of each tank (30 per treatment) were dissected into five macroareas (telencephalon, thalamus, cerebellum, brainstem and optic lobes) and immediately frozen by immersion in liquid nitrogen (frozen in at − 80 °C) for further analysis of monoaminergic activity. Dissected brain areas were weighted, homogenized and processed. Aliquots from the filtered supernatant was filtered were injected into the HPLC system on a reversed-phase condition with a phosphate based mobile phase with electrochemical detection (see^24^ for processing details and HPLC condition specifications). The concentrations of the compounds in a given sample were calculated by interpolating the corresponding peak height into a parallel standard curve using the Waters Breeze software. This method enables to quantify in only one chromatographic run the brain pool of serotonine (5-HT), dopamine (DA) and noradrenaline (NA) primarily stored within neurons; levels of their precursors (5-hydroxytryptophan; 5-HTP, and dihydroxyphenylalanine; DOPA), and some of their metabolites (which can reveal recent use of these neurotransmitters)^24^.

Additionally, whole brain samples were obtained from 12 fish across the three tanks of each treatment, and immediately immersed in liquid nitrogen (frozen in at − 80 °C) for antioxidant activity analyses. Whole fish brain samples (n = 24) were homogenized in 10 volumes (w/v) of 100 mM Tris–HCl buffer pH 7.5. Each homogenate was briefly sonicated (2–3 s), centrifuged at 9,000 × g at 4 °C for 10 min^25^ and supernatants collected and immediately frozen and stored at − 80 °C. All results were referred to the total protein content of the samples (Biorad Protein Assay) using bovine serum albumin as standard. CAT activity (K (s − 1)/mg protein) was measured by the method of Aebi^26^ based on the decomposition of H2O2 and monitored at 240 nm. SOD activity (mmol/s/mg protein) was determined by the degree of inhibition of the reduction of cytochrome C by superoxide anion generated by the xanthine oxidase/hypoxanthine system and recorded at 550 nm^27^. GPx activity (mmol/s/mg protein) was measured using an adaptation of the method of Flohé and Günzler^28^ with H2O2 as substrate. The decrease in NADPH absorbance at 340 nm during the oxidation of NADPH to NADP + , was indicative of GPx activity. GRd activity (mmol/s/mg protein) was measured by a modification of the Goldberg and Spooner^29^ method, in which the rate of conversion of GSSG to GSH was estimated by monitoring oxidation of NADPH in the assay system at 340 nm. AChase activity (µmol/min/mg protein) was determined by an adaptation of the method of Ellman et al.^30^ using thiocholine as a substrate. The product of hydrolysis by AChase is combined with 5, 5 dithiobis-2-dinitrobenzoic acid (DTNB) acid and monitored at 412 nm^31^. All antioxidant enzyme activities were determined with a Shimadzu UV-2100 spectrophotometer at 25 °C. MDA levels, as marker of lipid peroxidation, was analysed by a colorimetric assay specific for MDA. Briefly, samples or standards were placed in glass tubes containing n-methyl-2-phenyl-indole (10.3 mM) in acetonitrile:methanol (3:1). Then, HCl (12 N) was added, and the samples were incubated for 1 h at 45 °C. The absorbance was measured at 586 nm in a microplate spectrophotometer (Bio-Tek, PowerWaveXS)^31^. MDA concentration was calculated using a standard curve of known concentration.

### Statistical analyses

Multivariate general linear model (GLM, Type III, α = 0.095) and post-hoc Tukey test (α = 0.095) were used to assess differences between treatments (fixed factor) and tanks (nested factor) regarding the biometric parameters (response variables). Behavioural differences between treatments and over time were tested with repeated measures two-way ANOVA with treatment (EE vs. NE) as fixed factor and time (4 days) as the random factor. For neurophysiological analyses, fish from different tanks were pooled under treatment (EE vs. NE) level due to the low number of samples per tank. Then, multivariate general linear model (GLM, Type III, α = 0.095) and post-hoc Tukey test (α = 0.095) were used to assess differences between treatments (EE vs. NE; fixed factor) regarding enzymatic activities (CAT, SOD, GPx, GRd and AChase) and MDA levels in brain, but also monoamines levels in different regions of the seabream brain. Levene´s tests were applied to assess homogeneity of variances. The statistical package IBM-SPSS 20.0 (https://www.ibm.com/support/pages/node/723799) was used for all analyses.

## Results

Morphological variables at the beginning (t_0_) and at the end of the experiment (t_60_), as well as their increments, did not show any significant difference (GLM; *p-*value > 0.05) between treatments and tanks (see Supplementary Materials, Table S1). At the beginning of the experiment, no statistical differences were found between treatments regarding standard length (SL_0_: EE = 9.3 ± 0.1 cm, NE = 9.4 ± 0.1 cm; F = 0.633, *p-*value = 0.675), total weight (TW_0_: EE = 22.1 ± 0.8 g, NE = 21.8 ± 0.6 g; F = 0.559, *p-*value = 0.731) and Fulton´s condition index (K_0_: EE = 2.66 ± 0.03, NE = 2.61 ± 0.02; F = 1.104, *p-*value = 0.366). Similarly, no statistical differences were found at the end of the experiment between treatments regarding SL_60_ (EE = 13.2 ± 0.2 cm, NE = 13.0 ± 0.2 cm; F = 1.548, *p-*value = 0.185), TW_60_ (EE = 55.7 ± 2.2 g, NE = 54.4 ± 2.1 g; F = 1.504, *p-*value = 0.393) and K_60_ (EE = 2.43 ± 0.06, NE = 2.46 ± 0.03; F = 2.166, *p-*value = 0.060). Regarding the estimated increments of previous parameters, no statistical differences were found between treatments on ΔSL (EE = 3.7 ± 0.1 cm, NE = 3.6 ± 0.1 cm; F = 1.572, *p-*value = 0.178), ΔTW (EE = 33.3 ± 1.3 g, NE = 32.6 ± 1.3 g; F = 1.437, *p-*value = 0.221) and ΔK (EE = -0.16 ± 0.03, NE = -0.14 ± 0.04; F = 1.233, *p-*value = 0.307).

### Behavioural effects of enriched environments

**Figure 2:**
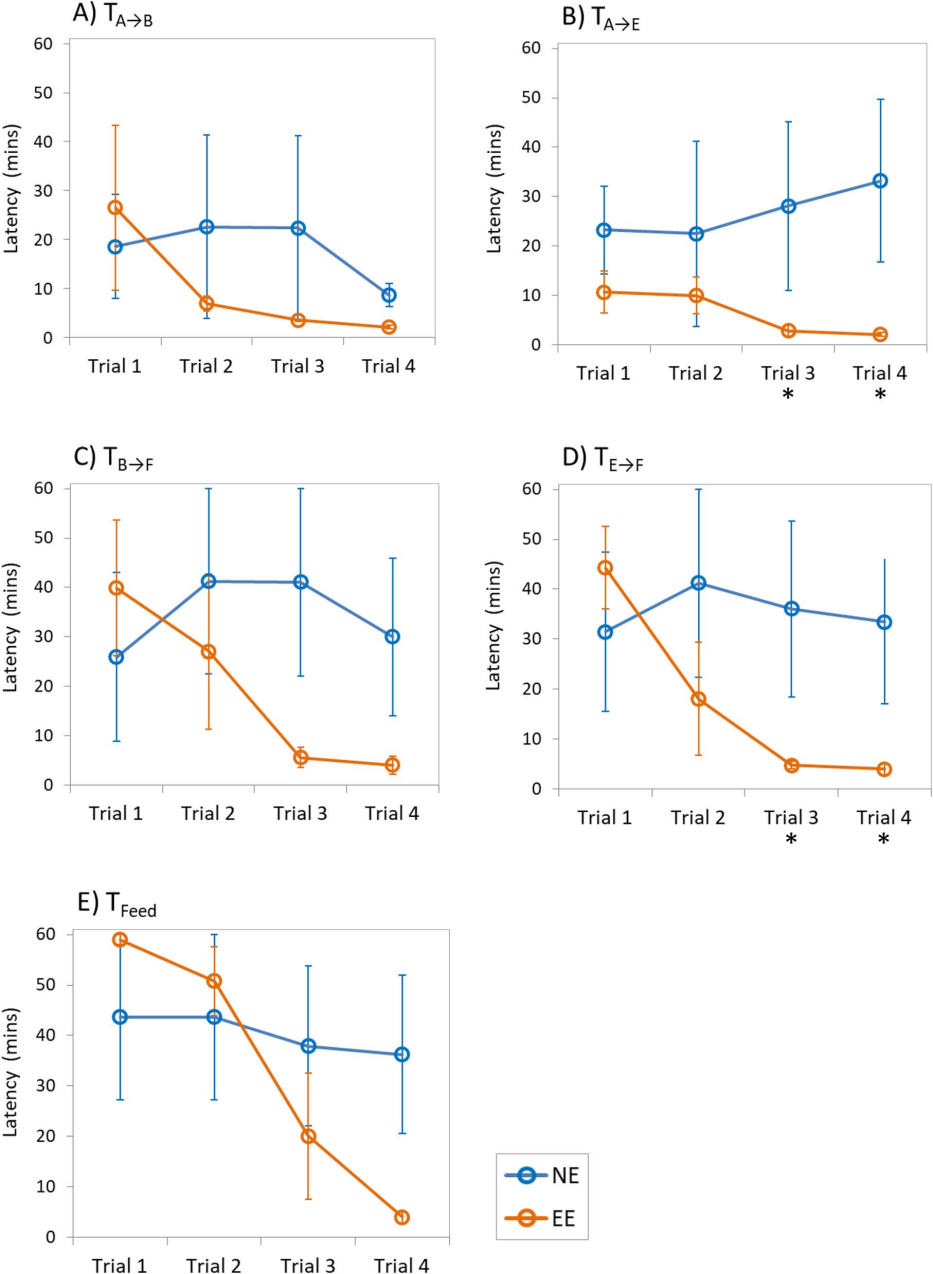
Mean latency (± SE) to enter in each maze area throughout the experimental trials. NE: Seabream reared under non-enriched environmental conditions (blue); EE: Seabream reared under environmental enriched conditions (orange). Statistical significance (*p-*value): * < 0.05.

**Figure 3:**
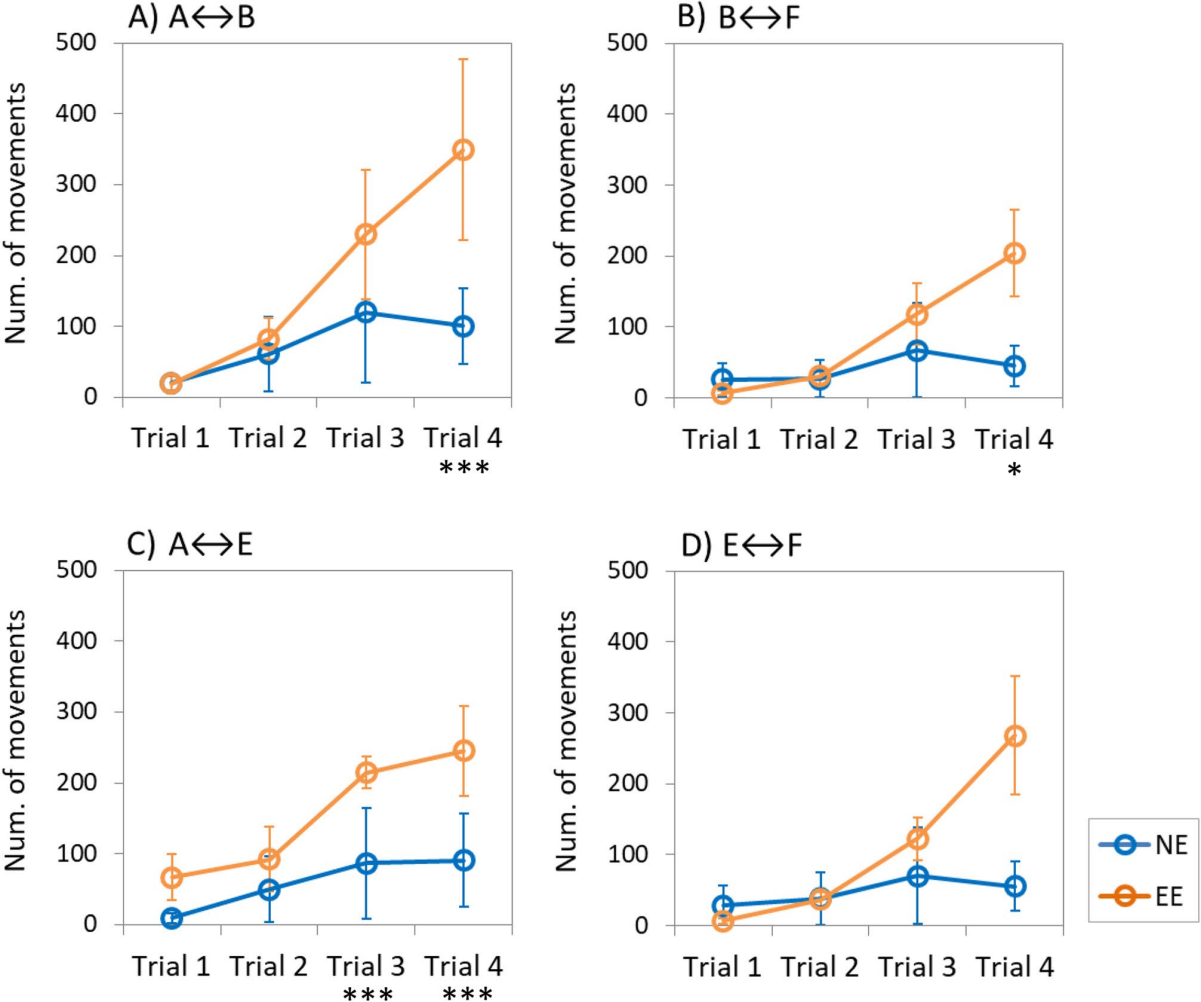
Mean number of movements (± SE) between areas (both ways) throughout the experimental trials. NE: Seabream reared under non-enriched environmental conditions (blue); EE: Seabream reared under environmental enriched conditions (orange). Statistical significance (*p-*value): * < 0.05, *** < 0.001.

Fish reared with EE showed an exponential decrease in the latency to move from area A to area B (T_A-B_) and a sharp decrease in time to move from B to F (T_B-F_) throughout the trials, although no significant differences were observed between treatments during trials in both cases (T_A-B_: F = 0.267, *p-*value = 0.267; T_B-F_: F = 4.212, *p-*value = 0.074; Fig. 2a,b). However, the latency to move from area A to E (T_A-E_), and the time to appear in area F from E (T_E–F_) was significantly lower in EE-reared fish during the last trials compared to seabream in NE (T_A-E_: F = 6.028, *p-*value = 0.040; T_E–F_: F = 6.105, *p-*value = 0.039; Fig. 2c,d). Regarding the latency of the first individual to bite the food in area F (T_Feed_), EE-reared fish showed a tendency to spend less time to bite in subsequent trials compared to NE-reared fish that showed no variation over time, although no significant differences were detected (T_Feed_: F = 0.942, *p-*value = 0.360; Fig. 3e). The total amount of fish movements recorded between area A and B (N_A↔B_), and between area B and F (N_B↔F_), were significantly higher in EE fish during last trial compared to NE fish in both cases (N_A↔B_: F = 11.673, *p-*value = 0.009; N_B↔F_: F = 6.663, *p-*value = 0.033)(Fig. 3a,b). Similarly, the number of movements observed between areas A and E (N_A↔E_) was significantly higher on EE-reared fish compared to NE-reared fish during the last trials (N_A↔E_: F = 29.251, *p-*value = 0.001; Fig. 3c). However, no significant differences were observed between treatments on the number of movements between areas E and F (N_E↔F_: F = 3.414, *p-*value = 0.102; Fig. 3d).

### Effects of EE on monoaminergic activity of fish brain regions

Main results from monoamine neurotransmitters, precursors and metabolites analyses in different brain regions can be found in Supplementary Materials (Table S2). In telencephalon, significant statistical differences were only obtained in DOPAC between treatments (F = 8.522, *p-*value = 0.007), with higher DOPAC levels in seabream from EE conditions (101.9 ± 18.6 pg g^−1^) compared to NE fish (48.2 ± 6.73 pg g^−1^). No significant differences (*p* > 0.05), however, were observed between treatments on the rest of monoamines analysed in telencephalon. Regarding monoamine activity levels in cerebellum, significant statistical differences were only obtained in 5-HT between treatments (F = 4.851, *p-*value = 0.034), resulting higher 5-HT levels in seabream from EE conditions (722.5 ± 215.4 pg g^−1^) compared to NE-reared fish (171.5 ± 82.2 pg g^−1^). No significant differences (*p* > 0.05), however, were observed between treatments regarding the rest of monoamines analysed in cerebellum. Similarly, no significant differences (*p* > 0.05) were detected between treatments in thalamus, brainstem and optic lobes on analysed monoamine levels. Similarly, DOPAC/DA and 5-HIAA/5-HT ratios did not differ (*p* > 0.05) between treatments in any of the brain areas analysed.

### Effects of EE on brain antioxidant enzymatic activity

**Figure 4:**
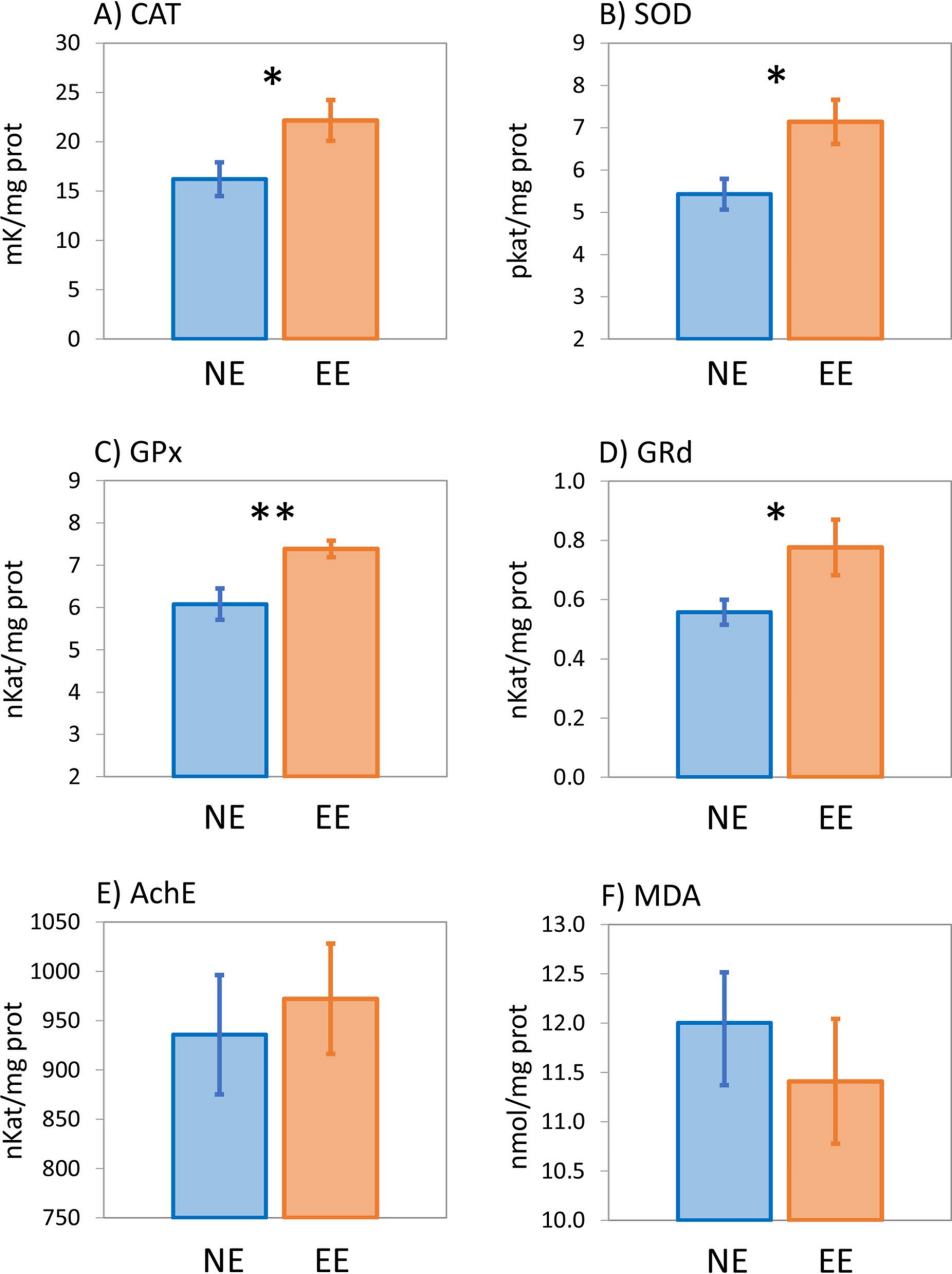
Activity levels of biomarkers of oxidative stress (mean values ± SE) measured in seabream brains under two different treatments (*NE* non-enriched, *EE* environmental enriched). *CAT* catalase, *SOD* superoxide dismutase, *GPX* glutathione peroxidase, *GRd* glutathione reductase, *AchE* acetylcholinesterase, *MDA* malondialdehyde. Statistical significance (*p*-value): * < 0.05, ** < 0.01.

The presence of EE in experimental tanks showed statistical differences in enzymatic activity of seabream with respect to NE environments (Fig. 4). Average (± SE) levels of brain CAT activity showed significant differences between treatments, resulting higher CAT levels in seabream under EE conditions than in NE-reared fish (EE: 22.73 ± 3.23 mK mg^−1^; NE: 16.22 ± 2.17 mK mg^−1^; F = 4.903, *p-*value = 0.037). Significant higher levels of SOD activity were also detected in seabream brain under EE conditions compared to NE treatment (EE: 7.14 ± 0.36 pkat/mg^−1^; NE: 5.42 ± 0.52 pkat/mg^−1^; F = 7.173, *p-*value = 0.014). Similarly, significant differences between treatments were also detected on GPx (EE: 6.78 ± 0.19 nmol mg-1; NE: 6.28 ± 0.28 nmol mg-1; F = 9.745, *p-*value = 0.005) and GRd (EE: 0.76 ± 0.25 nmol mg-1; NE: 0.55 ± 0.03 nmol mg-1; F = 4.546, *p-*value = 0.044) analysed, resulting higher activity levels in brains of EE-reared fish compared to NE-reared fish brains in both cases. No statistical differences between treatments however were found regarding activity levels of AChase (EE: 972.28 ± 38.76 nKat mg^−1^; NE: 935.84 ± 41.85 nKat mg^−1^; F = 0.195, *p-*value = 0.663) and MDA activity (EE: 11.41 ± 0.43 nmol mg^−1^; NE: 12.01 ± 0.35 nmol mg^−1^; F = 0.529, *p-*value = 0.475).

## Discussion

This study evidenced for the first time that structural environmental enrichment enhances welfare status of captive seabream, influencing positively on cognitive processes, behavioural responses, and brain-physiological functions. The deliberate addition of simple enrichment structures in the experimental rearing environment enhanced the spatial cognition and exploratory behaviour of juvenile seabream. Cognition is not a single process but rather consists of three interacting aspects: perception, learning, and memory^32^. Fish reared under enrichment are exposed to environmental challenges, higher visual complexity and new sensory stimulations. Results from the maze trials showed that EE-reared fish had better learning skills than the NE group but also that the experience is retained and consolidated through memory processes. The early development of fish brains is shaped by experiences with the environment and this can promote both neural plasticity and cognitive ability^33^. A previous study on rainbow trout (*Oncorhynchus mykiss*) indicates that the presence of stable stones and plastic plants in rearing aquaria increased fish exploratory behaviour and less intraindividual behavioural variation^10^. The provision of in-tank structures on Atlantic cod (*Gadus morhua*) increased their behavioural flexibility and social learning^8,9,11^. Similarly, a study on Atlantic salmon (*Salmo salar*) demonstrated that introducing temporally variable structural EE had positive effects on learning ability in the context of escaping a maze, where EE-reared fish were faster at finding their way out of a four-armed maze than individuals reared in standard, non-enriched hatchery conditions^12^. Our results also showed that EE-reared fish presented higher behavioural flexibility and lower latency to reach the food chamber compared to NE-reared fish. This ability to learn and remember spatial cues may increase, for example, the foraging success by remembering the location of better feeding grounds; or the chance for survival by remembering appropriate shelter locations in the presence of predators.

Different behavioural responses between treatments observed in this study may be associated with changes in monoaminergic activity in telencephalon. The teleost telencephalon plays a key role in spatial cognition^32,34^, processing complex spatial information to provide map-like representations that can help to locate specific places^35^. However, the fact that neither DA nor DOPAC/DA ratio differed between treatments may indicate that the activity peak had passed when the sampling was performed, which highlights the importance of timing when testing for neurotransmitters. Nevertheless, higher levels of DOPAC should correlate with recent peaks in DA^36^. Dopaminergic activity in different brain regions appears to influence locomotor activity, learning, motivation and social reward, but also represent differences on aggressive/avoidance or stress-reaction behaviours at fish individual level, when they achieve dominant status or higher social experience (e.g.^17,37–39^.). On the other hand, the serotonergic system has also a key role in neuronal plasticity^40^, influencing locomotor behaviour, cognitive processes, and stress responsiveness^16,41–43^. Although the 5-HIIA/5-HT ratio did not differ between treatments, the EE group showed higher 5-HT levels in cerebellum, which suggests increased serotonergic activity in this macroarea. Classical studies report the cerebellum being involved in motor coordination and control, but also link this region to more complex functions such as spatial memory in teleosts^44,45^. Our results seem to point in the same direction: the experimentally increased spatial complexity in the EE treatment elicited a better performance in tasks requiring spatial memory while also putatively enhancing the neuronal network in the cerebellum through the serotonergic system. It is interesting that other studies reported changes in behaviour yet different patterns in brain monoamine levels in gilthead seabream when exposed to EE, in that case tank substrate of different colours. The presence of blue or red-brown substrate on tank bottom resulted in suppression of aggressive behaviour compared to green substrate and no-substrate tanks^46^, but also decreased the serotonergic activity (5-HIAA/5-HT)^47,48^. Other work on seabream alevines reported a decrease of aggressiveness within a population when adding ropes as structural EE (similar to this study), as well as changes in the fish distribution in the tank, but brain monoaminergic activity was similar between EE and NE reared fish^20^. Several factors may account for these differences: (1) in the substrate study, the enrichment factor did not interfere with movement and swimming patterns. This may be determinant in which brain regions and neurotransmitters are activated; (2) In both examples, the search for differences in 5-HT metabolites was performed in whole-brain samples. It is therefore difficult to compare the present results with those from the mentioned papers. Overall, although the analysis of tissue concentrations of neurotransmitter metabolites does not reflect instantaneous neural activity, increased metabolite levels are in most cases considered an indicator of increased monoamine utilisation^49^.

Changes in exploratory and swimming behaviour responding to enriched and novel environments were well reflected on antioxidant enzyme activities measured in seabream brains. Higher swimming activity or stressful conditions may be related to a higher ventilatory activity, to ensure the supply of oxygen at the exact rate required by cellular oxygen metabolism, which is regulated primarily to avoid oxidative stress at the cellular level by antioxidant enzymes^19^. EE-reared seabream presented higher CAT, SOD, GPx and GRd levels compared to NE-reared fish, as a consequence of higher oxygen consumption, whereas MDA levels were similar between treatments (lipid peroxidation was not increased), suggesting that the EE group was better protected from oxidative cell damage. Therefore, EE environments seem to promote antioxidant enzyme activity on juvenile seabream, without causing oxidative damages, which may be a consequence of higher physical activity under healthy levels. Aspects such a locomotor behaviour and environmental conditions have been previously reported to influence antioxidant defences of fish. A previous work^50^ made comparative studies in different tissues of marine teleosts with different swimming activity, and reported greater SOD enzyme content in liver, heart and blood compared with slower-swimming species, which was correlated with higher metabolic oxygen consumption. Regarding seabream, several works demonstrated positive correlations of antioxidant enzyme activities in liver with fish behaviour (lethargy, social interactions) and stress (food restrictions, handling, transportation)^51–56^. It can be suggested, therefore, that the activity of antioxidant enzymes, as well as the concentration of MDA, are good candidates as biomarkers of stress, swimming exercise and welfare of fish. This adaptive response of cells and organisms to an increase of oxygen demand due to an increase of swimming exercise or physical stress can be translated as an “hormetic response”, where proteins like antioxidant enzymes are involved^57^. Hormesis is the phenomenon in which low doses or severities of a challenge cause one response while at a higher level or severity cause a response in the opposite direction, positive to negative, or vice versa^58^. The physical fitness of fish can be enhanced by low levels of stress or moderate challenges, such as enriched/novel rearing environments, while they can be reduced by elevated levels or prolonged durations of stress^59^. Psychological and physical conditioning involving low levels of stress, yet sufficient to activate the hypothalamus-pituitary-internal axis, can enhance stress-resistance in fishes (see^60^ for review); one can think of this as “stress hardening”^59^.

This study, therefore, shows that EE improved the welfare status of juvenile seabream. The addition of structural complexity to rearing tanks of a species that is adapted to such complexity in their natural environment is highly beneficial (see^60^ for a review), yet in fish farming this is surprisingly under-implemented. Our study demonstrated that EE are not beneficial but also not detrimental to the growth of the fish and might be used safely by the aquaculture industry. In addition, we provided evidence that EE “ticks all the boxes” from nature-based, function-based and feelings-based approaches towards fish welfare^3,61^: it provides opportunity for the expression of natural behaviours, improves physiological parameters and elicits the secretion of neurotransmitters associated with positive mental states. Ethologically relevant EE may therefore serve a broader purpose to fulfil needs that are not met under standard rearing conditions^3^. Given the advanced mental capacities of fish^62^, it is ethically^63^ and even legally^64^ imperative to provide these animals with appropriate rearing conditions. The application of measures that follow a positive welfare rationale^4^, rather than mere mitigation of negative effects is probably far more effective and beneficial for the industry, the consumer and most of all the fish^2^. Although physical structures might be a feasible, passive and non-invasive tool to improve welfare of captive fish, further works are needed in relation to structural design, husbandry conditions and fish developmental stage, as well as in other species, to be assessed under the production conditions in aquaculture.

## Supplementary information

Supplementary file1 (DOCX 21 kb)
