# Supplementary material for: Enriched environments enhance cognition, exploratory behaviour and brain physiological functions of Sparus aurata
Source: Sci Rep. 2020 Jul 9;10:11252. doi: 10.1038/s41598-020-68306-6 (PMC7347547; doi:10.1038/s41598-020-68306-6)
Supplement: Supplementary file 1 — Supplementary file1 (DOCX 21 kb) [file 41598_2020_68306_MOESM1_ESM.docx]

**Enriched environments enhance cognition, exploratory behaviour and brain physiological functions of *Sparus aurata***

Arechavala-Lopez, P.^1,2,*^, Caballero-Froilán, J.C.^3^, Jiménez-García, M.^3^, Capó, X^4,5^, Tejada S^3,5^, Saraiva, J.L^1^, Sureda, A.^4,5^, Moranta, D.^3^

**Supplementary Materials**

**Table S1.** Average values (±SE) of body length (SL; cm), body weight (TW; g) and Fulton´s condition factor (K) of seabream at the begging (t_0_) and at the end of the experiment (t_60_), as well as estimated respective increments (Δ) of juvenile seabream kept in tanks under experimental enriched (EE) and non-enriched (NE) conditions during 60 days.

| **Treat.** | **Tank** | **N** | **S.L._0_** | **T.W._0_** | **K_0_** | **S.L._60_** | **T.W._60_** | **K_60_** | **ΔS.L.** | **ΔT.W.** | **ΔK** |
| --- | --- | --- | --- | --- | --- | --- | --- | --- | --- | --- | --- |
| **EE** | 2 | 15 | 9.4 (±0.2) | 22.7 (±1.3) | 2.66 (±0.04) | 13.3 (±0.2) | 59.1 (±2.3) | 2.48 (±0.04) | 3.7 (±0.1) | 35.5 (±1.4) | -0.15 (±0.04) |
|  | 3 | 15 | 9.2 (±0.2) | 20.9 (±1.4) | 2.65 (±0.04) | 12.9 (±0.2) | 53.5 (±2.1) | 2.49 (±0.02) | 3.7 (±0.1) | 32.6 (±1.1) | -0.16 (±0.03) |
|  | 6 | 15 | 9.5 (±0.2) | 23.1 (±1.4) | 2.67 (±0.04) | 13.3 (±0.3) | 54.6 (±2.1) | 2.31 (±0.09) | 3.5 (±0.1) | 31.6 (±1.2) | -0.17 (±0.03) |
|  | Total | 45 | 9.3 (±0.1) | 22.1 (±0.8) | 2.66 (±0.03) | 13.2 (±0.2) | 55.7 (±2.2) | 2.43 (±0.06) | 3.7 (±0.1) | 33.3 (±1.3) | -0.16 (±0.03) |
| **NE** | 1 | 15 | 9.5 (±0.1) | 22.7 (±1.1) | 2.64 (±0.04) | 13.3 (±0.2) | 57.2 (±2.3) | 2.43 (±0.01) | 3.8 (±0.1) | 34.5 (±1.7) | -0.21 (±0.03) |
|  | 4 | 15 | 9.2 (±0.2) | 20.7 (±1.1) | 2.62 (±0.04) | 12.7 (±0.1) | 52.3 (±1.8) | 2.53 (±0.04) | 3.4 (±0.1) | 31.4 (±1.1) | -0.08 (±0.04) |
|  | 5 | 15 | 9.4 (±0.2) | 21.9 (±1.2) | 2.56 (±0.03) | 13.1 (±0.2) | 53.9 (±2.2) | 2.41 (±0.03) | 3.6 (±0.1) | 32.1 (±1.2) | -0.14 (±0.04) |
|  | Total | 45 | 9.4 (±0.1) | 21.8 (±0.6) | 2.61 (±0.02) | 13.0 (±0.2) | 54.4 (±2.1) | 2.46 (±0.03) | 3.6 (±0.1) | 32.6 (±1.3) | -0.14 (±0.04) |

**Table S2.** Average values (±SE) of monoamine levels (pg g^-1^) analysed in different regions of the brain of seabream kept under non-enriched (NE; n=19) and enriched (EE; n=19) conditions in experimental tanks during 60 days. Statistical significant difference (p<0.05) are marked in bold and different superscript letters.

|  | **Telencephalon** | |  | **Thalamus** | |  | **Cerebellum** | |  | **Brainstem** | |  | **Optic Lobes** | | |
| --- | --- | --- | --- | --- | --- | --- | --- | --- | --- | --- | --- | --- | --- | --- | --- |
|  | **NE** | **EE** |  | **NE** | **EE** |  | **NE** | **EE** |  | **NE** | **EE** |  | **NE** | **EE** |  |
| **NA** | 701.7 (±70.6) | 811.2 (±162.9) |  | 683.3 (±80.9) | 624.0 (±43.9) |  | 176.1 (±18.6) | 295.9 (±60.7) |  | 712.5 (±38.8) | 686.6 (±26.3) |  | 470.9 (±57.5) | 479.6 (±61.8) |  |
| **DA** | 116.4 (±15.3) | 127.2 (±19.6) |  | 148.7 (±17.7) | 142.0 (±10.8) |  | 94.6 (±13.4) | 119.1 (±13.9) |  | 135.1 (±21.9) | 112.3 (±15.3) |  | 37.7 (±9.0) | 28.4 (±6.5) |  |
| **DOPA** | 141.7 (±29.1) | 189.4 (±42.1) |  | 150.9 (±11.1) | 171.8 (±17.9) |  | 220.7 (±28.2) | 190.6 (±26.5) |  | 105.1 (±12.4) | 76.5 (±8.2) |  | 24.3 (±5.2) | 21.1 (±5.1) |  |
| **DOPAC** | **48.2 (±6.73)^a^** | **101.9 (±18.6)^b^** |  | 58.3 (±9.8) | 68.8 (±11.8) |  | 63.2 (±15.2) | 59.9 (±10.3) |  | 6.45 (±1.9) | 11.25 (±2.7) |  | 26.6 (±4.1) | 34.6 (±5.0) |  |
| **5-HT** | 2464.4 (±337.1) | 2012.3 (±346.5) |  | 2533.8 (±209.6) | 2015.6 (±156.6) |  | **171.5 (±82.2)^b^** | **722.5 (±215.4)^a^** |  | 845.4 (±62.1) | 825.7 (±49.0) |  | 431.8 (±39.9) | 439.8 (±32.8) |  |
| **5-HTP** | 350.8 (±73.2) | 286.8 (±49.6) |  | 149.0 (±19.0) | 169.5 (±15.2) |  | 151.5 (±44.8) | 99.1 (±26.5) |  | 105.2 (±8.2) | 121.3 (±5.5) |  | 69.5 (±5.9) | 66.5 (±4.0) |  |
| **5-HIAA** | 157.3 (±59.5) | 106.6 (±48.3) |  | 158.0 (±11.3) | 149.7 (±13.4) |  | 285.2 (±69.6) | 271.7 (±68.2) |  | 324.9 (±114.9) | 334.7 (±155.2) |  | 44.3 (±5.7) | 39.8 (±2.9) |  |
| **DOPAC/DA** | 0.48 (±0.06) | 1.04 (±0.34) |  | 0.55 (±0.14) | 0.59 (±0.18) |  | 0.77 (±0.11) | 0.71 (±0.09) |  | 0.15 (±0.03) | 0.17 (±0.03) |  | 1.66 (±0.78) | 1.82 (±0.38) |  |
| **5-HIAA/5-HT** | 0.08 (±0.04) | 0.06 (±0.01) |  | 0.11 (±0.03) | 0.09 (±0.02) |  | 1.84 (±1.17) | 0.76 (±0.36) |  | 0.40 (±0.15) | 0.44 (±0.24) |  | 0.11 (±0.02) | 0.10 (±0.01) |  |
